# A randomized controlled trial comparing two types of pneumatic compression for breast cancer-related lymphedema treatment in the home

**DOI:** 10.1007/s00520-012-1455-2

**Published:** 2012-05-02

**Authors:** Caroline E. Fife, Suzanne Davey, Erik A. Maus, Renie Guilliod, Harvey N. Mayrovitz

**Affiliations:** 1 Department of Internal Medicine, Division of Cardiology, University of Texas Health Science Center, Memorial Hermann Center for Wound Care and Lymphedema Management, 6431 Fannin, MSB 1.247, Houston, TX 77030 USA; 2 Healing Hands of Lymphatics, Hallandale Beach, FL USA; 3 College of Medical Sciences, Nova Southeastern University, Ft. Lauderdale, FL USA

**Keywords:** Lymphedema, Pneumatic compression devices, Breast cancer, Manual lymphatic drainage, Flexitouch® system, Bio compression 2004 sequential circulator

## Abstract

**Purpose:**

Pneumatic compression devices (PCDs) are used in the home setting as adjunctive treatment for lymphedema after acute treatment in a clinical setting. PCDs range in complexity from simple to technologically advanced. The objective of this prospective, randomized study was to determine whether an advanced PCD (APCD) provides better outcomes as measured by arm edema and tissue water reductions compared to a standard PCD (SPCD) in patients with arm lymphedema after breast cancer treatment.

**Methods:**

Subjects were randomized to an APCD (Flexitouch system, HCPCS E0652) or SPCD (Bio Compression 2004, HCPCS E0651) used for home treatment 1 h/day for 12 weeks. Pressure settings were 30 mmHg for the SPCD and upper extremity treatment program (UE01) with standard pressure for the APCD. Thirty-six subjects (18 per group) with unilateral upper extremity lymphedema with at least 5% arm edema volume at the time of enrollment, completed treatments over the 12-week period. Arm volumes were determined from arm girth measurements and suitable model calculations, and tissue water was determined based on measurements of the arm tissue dielectric constant (TDC).

**Results:**

The APCD-treated group experienced an average of 29% reduction in edema compared to a 16% increase in the SPCD group. Mean changes in TDC values were a 5.8% reduction for the APCD group and a 1.9% increase for the SPCD group.

**Conclusion:**

This study suggests that for the home maintenance phase of treatment of arm lymphedema secondary to breast cancer therapy, the adjuvant treatment with an APCD provides better outcomes than with a SPCD.

## Introduction

Lymphedema is a condition resulting from lymphatic dysfunction in which persistent swelling exists due to an abnormal accumulation of protein-rich fluid in an extremity or other body region and is accompanied by marked subcutaneous and skin changes as the condition worsens [1]. Primary lymphedema is caused by the abnormal development of the lymphatic system and may present at birth, during late adolescence, or in early adulthood. Secondary lymphedema results from extrinsic damage or obstruction of the lymphatics. In developed countries, breast cancer treatment is recognized as the major cause of secondary lymphedema [1–3]. The incidence of breast cancer-related lymphedema rises dramatically from 3 to 15% after sentinel node biopsy, to 10–20% after complete axillary dissection, and 30 to 50% with subsequent radiotherapy [4–7]. Recent data obtained from fluorescent lymphatic imaging suggests that some patients developing secondary lymphedema may have had a genetic predisposition to lymphatic malfunction [8, 9]. Currently, no cure exists for lymphedema, so treatment aims to ameliorate the swelling, lessen its severity and symptoms, and impede progression. Effective long-term treatment strategies are, therefore, crucial.

The traditional initial treatment for lymphedema is complete decongestive therapy (CDT), which consists of manual lymphatic drainage (MLD), short-stretch compression bandaging, decongestive exercises, and skin care [10]. While this regimen works effectively for most patients when they are treated in a clinical setting by a well-qualified therapist, ultimately it is necessary for the patient to attempt to maintain the gains and manage this life-long condition in a home setting. Successful transfer to the home care setting is challenging. Many patients find the procedures, particularly those for self-MLD and bandaging, time-consuming and difficult to carry out, especially if they have physical limitations from comorbidities. Poor patient adherence to prescribed home treatments can result in failure to maintain progress and may likely negate gains achieved during previous clinical treatments.

One treatment adjunct that may aid the patient in the home management setting is use of pneumatic compression devices (PCDs). PCDs utilize an air compressor unit that attaches to a garment or series of garments. The garment chambers sequentially inflate and deflate applying pressure against the skin thereby creating a treatment effect. PCDs range from simple single-chamber or multichamber devices with limited adjustability, to advanced devices with more garment options and a wide array of treatment options and adjustability to address different clinical needs such as fibrosis, truncal swelling, chronic wounds, or localized swelling. The adjustability and expanded programs available with many advanced PCDs (APCDs) provide for faster and more succinct cycles of pressure and relaxation with compression levels better approximating those applied with MLD [11]. Some APCDs provide specific garments enabling truncal treatment, which is deemed fundamental in clinical training in MLD technique and is based on lymphatic architecture and animal experimental evidence [11–14].

Validation of treatment and outcome advantages associated with advanced devices in comparison to standard devices is lacking. Although, several studies have reported positive results with use of PCD treatment [15–25], it is difficult to compare outcomes from the initial body of evidence regarding early PCDs with more current APCD options. Many of these studies are over 10 years old and lack sufficient detail about the devices studied to enable comparisons to aid in device selection or treatment protocol with regard to currently marketed devices, especially the newer PCD technologies [26]. Of significance, most of the treatment protocols studied were not practical or sustainable for long-term, as treatment durations ranged from 2 to 6 hours daily [26] and/or were limited to lower extremity lymphedema [19, 24]. One comparative study has been conducted to date regarding APCDs. A pilot RCT investigated the home maintenance phase of ten patients who had unilateral breast cancer-associated lymphedema of the arm by comparing self-MLD with an APCD for 1 h each day for 14 days followed by crossover to the other treatment with a 1-week washout period before each treatment [27]. Post-treatment arm volume and patients’ mean weight significantly decreased after using the APCD but not after self-administered MLD [27].

Despite development of some clinical consensus that APCDs may yield better outcomes for some patients than simple PCDs [28, 29], further published evidence is lacking. Based on the theoretical advantages potentially offered by an APCD as supported by prior literature, we hypothesized that an APCD would result in better outcomes than less advanced systems when used in the home environment. The specific goal of this study was to test this hypothesis by evaluating and comparing changes in arm edema volume and local tissue water content achieved with the use of either an APCD or standard PCD (SPCD) system.

## Materials and methods

This study was a prospective, randomized controlled trial in which patients were consented and randomized in a 1:1 ratio to either a SPCD (Bio Compression 2004 Sequential Circulator PCD, Bio Compression Systems, Moonachie NJ, USA) or an APCD (Flexitouch system, Tactile Systems Technology, Inc., Minneapolis MN, USA). Each device was to be used for the home maintenance phase of treatment for secondary upper extremity lymphedema caused by breast cancer treatment.

Three centers participated in this study: Healing Hands of Lymphatics (Hallandale Beach, FL, USA), Washington Hospital Healthcare System (Fremont, CA, USA), and Memorial Hermann Hospital (Texas Medical Center, Houston, TX, USA). The trial was approved by the following institutional review boards (IRBs): Sterling IRB (3139, 02-16-2009), Washington Hospital Healthcare System IRB (02-19-2009), and University of Texas IRB (HSC-MS-10-0050, 03-05-2009). This study was conducted in accordance with US and international good clinical practices (FDA Title 21, part 812 and International Conference on Harmonization guidelines), applicable government regulations and institutional research policies and procedures. Subjects who were recruited from the participating lymphedema treatment centers had either previously sought or were currently seeking treatment for their lymphedema. A total of 36 patients (equally divided between standard and APCD use) form the basis of the present report. These 36 patients were fully evaluated for edema volume changes after 12 weeks of in-home treatment and 28 of these patients (also equally divided) were evaluated for changes in arm tissue water content.

### Eligibility

Subjects had to be at least 18 years old and able to give informed consent. A confirmed diagnosis of upper extremity lymphedema secondary to breast cancer treatment was required, and each patient had to have previously completed intensive lymphedema treatment (phase I) and discharged to home maintenance (phase II) but the patient must not have received in-home PCD therapy for the past 3 months. Further, at least 5% edema volume needed to be present at the time of study enrollment.

### Baseline data

Demographic information including age, gender, race, arm dominance, and BMI was collected at baseline. Medical history data collected included type of surgery, chemotherapy, radiation, time since surgery, and number of nodes removed, if applicable. Table 1 summarizes these data for all subjects separated by randomly assigned groups (SPCD vs. APCD).

**Table 1:** Baseline comparison of the advanced and standard PCD groups (APCD, SPCD)

Characteristic	APCD^a^ (*n* = 18)	SPCD^b^ (*n* = 18)	*p* value
Age	63.9 ± 12.2	59.7 ± 12.6	0.315
BMI	28.2 ± 4.6	30.6 ± 7.4	0.258
Months between surgery and study start	105.9 ± 119.7	75.4 ± 73.4	0.815^c^
Number of nodes removed	11.9 ± 8.7	12.6 ± 11.2	0.987^c^
Surgery type			0.441
Mastectomy	12 (67)	15 (83)	
Lumpectomy	6 (33)	3 (17)	
Radiation therapy			0.717
Yes	12 (67)	13 (72)	
No	6 (33)	5 (28)	
Chemotherapy			0.675
Yes	15 (83)	14 (78)	
No	3 (17)	4 (22)	
Race			0.725
Caucasian	10 (56)	13 (72)	
African American	3 (17)	3 (17)	
Hispanic	2 (11)	1 (6)	
Other	3 (16)	1 (6)	
Affected arm			0.724
Left	13 (72)	11 (61)	
Right	5 (23)	7 (39)	
Surgery on dominant side	5 (28)	7 (39)	0.725

Numbers in parentheses are percentages

^a^Flexitouch system, HCPCS E0652

^b^Bio Compression 2004 Sequential Circulator, HCPCS E0651

^c^ *p*-value based on Mann–Whitney test

Tests for baseline group differences in age and BMI, number of nodes that were removed during the subjects’ initial surgery as well as time between surgery and study start showed no significant difference as evaluated via independent *t* tests (Table 1). Tests for normality of the data for the number of nodes that were removed during the subjects’ initial surgery as well as time between surgery and study start (Shapiro–Wilk test) indicated non-normality (*p* < 0.001), so this data was tested using the Mann–Whitney test. Results (Table 1) show insignificant differences in both parameters between groups. Further, tests for baseline group differences in race, affected arm side, dominant arm side, type of surgery, radiation treatment, and chemotherapy also showed no significant difference as evaluated via Pearson’s Chi Square analyses (Table 1). Thus, with respect to these demographic variables, the two groups were well balanced at entry into the study.

### Arm girth measurements

Arm circumferences (girths) were measured with a Gulick II tape measure. The Gulick II tape measure (model 67020; Country Technology, Inc., Gay Mills, WI) has a tension meter attached, so that the tape’s tension can be standardized during measurement. The no-stretch, retractable tape is calibrated to indicate precisely a 4-once tension. During measurement, the end of the tensioning mechanism is pulled until the calibration point, a silver disk separated by two colored beads, is seen. Girth measurements were done with subjects seated and their arms extended in front of them with their palm resting on their ipsilateral knee. Girths were measured at 4-cm intervals starting at the ulnar styloid and ending at the last full 4-cm interval near the axilla. Girth measurements were taken primarily by one person at each of the three sites. These individuals were all experienced lymphedema therapists who routinely perform these measurements daily in their active lymphedema practice. The girth values served as inputs to a software algorithm that calculates arm volume based on a standardized and validated frustum model [30–33]. Absolute arm volumes of affected arms (VA) and contralateral arms (VC) at week 0 and week 12 were determined from these girth measurements. Edema volumes in milliliters (EVOL) were calculated as (VA − VC) and percentage edema volume (%EVOL) was calculated as 100(VA − VC) / VC.

### Local tissue water

An index of local skin tissue water was measured using the tissue dielectric constant (TDC) method [34, 35]. The TDC is the electrical dielectric constant or permittivity of the tissue and is expressed relative to the permittivity of a vacuum, so it is a dimensionless number. Its measured value depends on the amount of water within the measurement volume; pure water has a value of about 78. With the probe and frequency used (300 MHz), the TDC value reflects both free and bound water within the measurement volume to a skin depth of about 2.5 mm. TDC values were obtained at weeks 0 and 12 using a commercial device (MoistureMeter-D, Delfin Technologies, Ltd., Kuopio, Finland). TDC measurements were made on both anterior forearms at a standardized site along the forearm midline located 8 cm distal to the antecubital crease. Triplicate measurements at each site were obtained by placing the probe in contact with the skin and held in position using gentle pressure. These triplicate measurements were averaged to produce a single value for each site. These measurements were made in 28 subjects (14 subjects in the APCD group and in 14 subjects in SPCD group) all coming from a single center, Healing Hands of Lymphatics (Hallandale Beach, FL), per protocol. Measurements were made at week 0 prior to the start of treatment and at week 12 at the end of treatment. This method for assessment has been extensively used to assess localized skin water and its change [34–38].

### Protocol and procedures

After consent and randomization, subjects were seen in the clinic for their baseline (week 0) measurements. Each subject was seen and examined by a study clinician who provided the subject with specific instructions to ensure thorough understanding of the operation of the device to which they were assigned. Subjects were reminded to continue the other components of their home care including exercise, skin care, and compression garment use as previously recommended by their clinician. Subjects were also instructed to complete the daily usage log pertaining to the assigned device and to document treatment compliance and were instructed on the treatment protocol.

### Treatment protocol

The assigned PCD was used for home treatment 1 h/day in addition to existing routine care, which included compression garment wear for 23 h per day. The SPCD consists of a gradient, sequential pneumatic compressor weighing 6 lbs with dimensions of 5.5″ × 12″ × 8″. It can be set within a pressure range of 0–125 mmHg. The compressor unit attaches to an arm garment with four compression chambers ranging from approximately 15 to 24 cm wide depending on garment size. Chambers inflate sequentially at a rate of 18 s/chamber until all chambers are fully inflated for a total inflation time of approximately 72 s. Full garment inflation is then held for 22 s before all chambers simultaneously deflate for 18 s. Garments have zipper closures and are made of 200 denier nylon-coated Oxford with 3 mils of polyurethane. The SPCD was set according to the manufacturer’s suggested setting of 30 mmHg.

The APCD consists of an electronic controller that attaches to three garments that treat the full upper extremity, which includes the arm, adjacent chest, and truncal quadrant. The controller unit weighs 8 lbs with dimensions of 10.4″ × 9.8″ × 4.7″. The garment set contains 26–28 (depending on garment size) narrow, curved chambers ranging from 3.8 to 4.4 cm wide which wrap around to follow the contours of the limb and trunk and close with hook and loop fasteners. The outer fabric of the garment is made of 100% nylon and is latex free. The inflation/deflation cycle for each chamber is 1–3 s in duration. No two chambers are fully inflated simultaneously. The APCD system has 13 therapy program options and applies light, variable pressure to the trunk and the affected arm using multichambered, inflatable, and stretchable fabric garments. The APCD was set to the full upper extremity program (UE01) at the device’s standard pressure setting according to the manufacturer’s suggested settings. Published data indicates that this standard setting applies between 9.0 ± 4.2 mmHg and 13.7 ± 4.8 mmHg to the forearm during the standard treatment program [39].

### Data analysis

The goal of the main study analysis was to evaluate possible differential effects of device treatment on edema volume and local tissue water. Possible differences between devices were assessed using a general linear model (GLM) for repeated measures (SPSS v13) with week (week 12 vs. week 0) as the within-subject repeated measure and device (standard or advanced) as the between-subjects factor. The significance level for the acceptance of a device effect was a priori set to a *p* value of 0.05 as determined for device–week interaction. The edema volume analysis included 18 subjects in the APCD group and 18 in the SPCD group. The local tissue water analysis included 14 subjects in each group. Possible differences in parameters between groups or differences between arms at week 0 and week 12 were separately tested using *t* tests for independent samples.

Additional endpoints that were evaluated were the number/severity of adverse events. Primary safety endpoints included any signs or symptoms of acute infection or other adverse clinical event defined as any symptom, sign, illness, or experience that developed or worsened in severity during the course of the study. Serious adverse events were defined as an event that is fatal, life threatening, requires hospitalization, or if left untreated could lead to persistent disability.

## Results

**Fig. 1:**
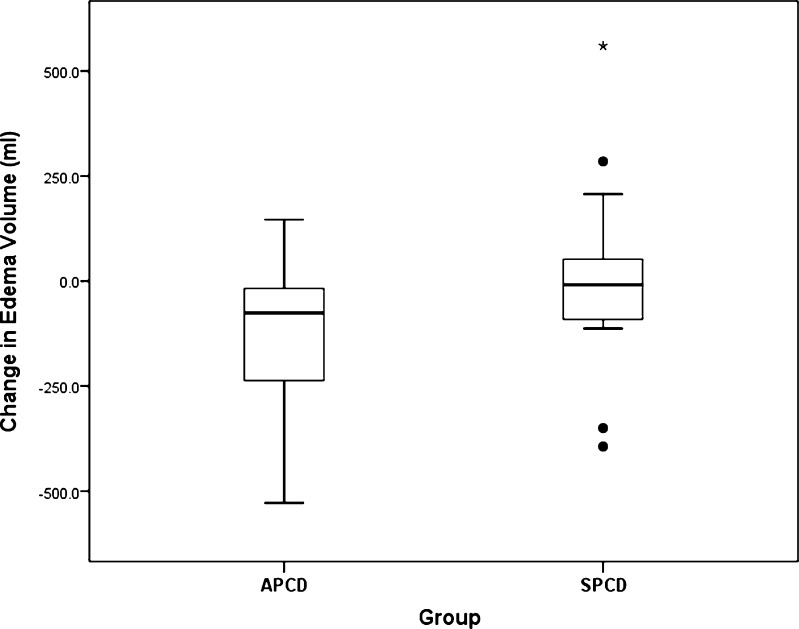
Changes in edema volume from week 0 to week 12 (*box plot*). *Circles* and *star* indicate possible outliers

Arm volumes, edema volumes, and tissue water results are given as mean ± standard deviation and summarized in Table 2. At baseline (week 0), there were no significant between-group differences in affected or control arm volumes, edema volume, or TDC values of affected or control arms. Contrastingly, and as expected, affected arms had significantly greater TDC values than control arms indicating greater skin tissue water of the affected arm. The GLM repeated measures analysis showed a significant group by week interaction for both edema volume and for affected arm TDC values but no interaction for TDC values of the control arm. This is consistent with a significant device treatment effect characterized by a week 0 to week 12 edema volume change of −118 ± 170 ml for the advanced group and +6.3 ± 216 ml change for the standard group. Figure 1 is a box plot showing these changes in edema volume from week 0 to week 12. Negative values denote reductions in edema volume. These absolute changes correspond to changes in percentage edema volume of −29 ± 44% for the advanced group vs. +16 ± 63% for the standard (*p* = 0.018). Repeated measures analysis also showed a significant group by week interaction for the affected arm TDC value with the advanced group demonstrating a reduction in TDC of −3.1 ± 4.9 as compared to +0.4 ± 3.9 for the standard group (*p* = 0.05). These absolute values correspond to a mean reduction in TDC value of 5.8% for the advanced group and a 1.9% increase for standard group.

**Table 2:** Volume and TDC result summary

Group	Week 0			Week 12			Week by group^b^
	APCD	SPCD	*p* value^a^	APCD	SPCD	*p* value^a^	
Affected arm volume (ml)	3,102 ± 755	3,104 ± 966	0.994	2,952 ± 724	3,013 ± 773	0.645	0.141
Control arm volume (ml)	2,546 ± 657	2,573 ± 695	0.902	2,514 ± 599	2,537 ± 631	0.912	0.942
Edema volume (ml)	556 ± 318	531 ± 372	0.823	438 ± 344	537 ± 293	0.363	0.050^*^
Percent edema volume (%)	23.0 ± 13.9	19.9 ± 11.9	0.471	18.2 ± 14.0	21.0 ± 10.7	0.571	0.047^*^
TDC of affected arm	36.9 ± 9.8	33.2 ± 7.8	0.260	33.8 ± 7.6	33.5 ± 6.6	0.904	0.049^*^
TDC of control arm	26.2 ± 3.8	25.3 ± 3.6	0.502	25.6 ± 2.4	25.8 ± 3.8	0.907	0.510

Data are mean ± standard deviation. Volume data based on 18 per group, and TDC data based on 14 per group

^a^As determined by independent *t* tests between groups

^b^Significance of week by group interaction

^*^Statistically significant

Device compliance was very good in both groups with the APCD group completing a total of 1,434 treatments out of 1,512 (95%) and the SPCD group completing 1,494 out of 1,512 (99%). Per patient report, compliance with the other home treatment components between the groups was very similar, with the biggest difference in exercise. In both groups, 78% (14 per group) reported compliance with daily compression garment wear with 22% (four per group) reporting partial compliance defined as garment wear less than 3 days/week or only during the night. Likewise, the same number in each group, 14 (67%), reported performing self-massage daily or almost daily, with 33% or six in each group reporting infrequent compliance with daily self-massage. Compliance with skin care was high and equal in both groups with 94% (17) in each group reporting daily compliance. Regarding exercise, 83% (15) of the SPCD group reported daily exercise compared to 61% (11) in the APCD group. It is important to note that subjects were instructed to continue with all other home treatment components they were doing prior to starting this study.

Seven adverse events were recorded that were classified as “unlikely,” “possibly,” or “definitely” device-related: one in the APCD group and six in the SPCD group. Five were further classified as “serious” and one “nonserious”. Table 3 provides the descriptions of these adverse events.

**Table 3:** Description of adverse events that might be device-related for advanced and standard PCD groups (APCD, SPCD)

Description	Group	Device-related	Category
Rash on arm	SPCD	Unlikely	Nonserious
Increased arm swelling	SPCD	Possibly	Serious
Breast inflammation; increased swelling and pain; developed infection and fibrosis	SPCD	Possibly	Serious
Increased hand swelling	SPCD	Definitely	Serious
Pain in forearm and numbness in fingers	SPCD	Definitely	Nonserious
Increased swelling of hand and torso; pain in axilla and back	SPCD	Definitely	Serious
Increased swelling of lymph nodes in contralateral axilla	APCD	Possibly	Serious

## Discussion

The results of this pilot study suggest that application of the APCD for the home maintenance phase of lymphedema secondary to breast cancer treatment provides better outcomes as judged by the significant arm edema and tissue water reductions for the APCD as compared to the SPCD with fewer complications. Because baseline subject characteristics were similar between groups and treatment time and compliance to treatment was essentially the same, we suggest that the outcome differences may be associated with differences between device function and treatment area.

The SPCD utilizes a slower inflation/deflation cycle wherein the garment takes approximately 72 s to fully inflate; inflation is held for 22 s and then the air is released from all chambers simultaneously. This process results in application of a static pressure to the full arm at a level likely above that which would compress lymphatic capillaries and thereby prevent lymph uptake during this interval [40–42].

The APCD utilizes a faster treatment cycle whereby each chamber inflates/deflates in 1–3 s before the next chamber inflates. This faster and more succinct cycle of pressure and release approximates those techniques applied with MLD [11] and would be consistent with a pressure profile that has been described to increase lymph drainage [11, 39]. The resulting dynamic and variable pressures likely induce the initial lymph capillaries to respond to the pressure changes occurring in the skin. Furthermore, the more rapidly changing pressure waves may better facilitate lymph drainage because this timing better corresponds to the timing of arterial and respiratory pulses, which are thought to stimulate lymphatics [11, 43, 44].

In addition, the garments of the APCD studied here provide specific directional movement that covers more of the affected region than does the SPCD, therefore treating more surface area, while following the lymphatic anatomy of the full upper extremity quadrant. A recently published pilot study utilizing near-infrared fluorescence imaging techniques supports the concept that this treatment approach may enhance lymphatic contractility. Adams et al. reported that significant increase in lymph vessel contractility was demonstrated with this same APCD [12].

Although the present findings demonstrated a statistically significant improvement in both edema volume and tissue water reductions in favor of the APCD, there remains the question as to whether the measured differences are of clinical import. Directly bearing on this question is the result of a recent study [45], in which it was concluded that even small limb volume changes have an impact on breast cancer survivors; as little as 5% volume differential was regarded as clinically significant with the frequency of signs and symptoms significantly increasing as limb volume increased. These signs and symptoms included tenderness, firmness/tightness, swelling, heaviness, and aching that ultimately translated into significant decrements in quality of life [45]. Although such signs and symptoms were not recorded in the present study, it is likely that the 29% average reduction in edema volume noted with subjects using the APCD would have produced symptom improvement that would likely have translated to quality of life improvements. Along with the small sample size, the absence of symptom burden, quality of life and functional outcomes in the present pilot study are limitations. However, the demonstration of the improved quantitative outcomes in edema volume and tissue water via APCD use suggests that further and more in-depth research is justified. This would include additional research with larger numbers of subjects and more comprehensive outcome measures with an ultimate goal of defining subject populations who would optimally benefit from the use of each type of PCD device without increased risk of complications.
